# Involvement of executive resources in prediction: Effects of load and hearing loss in older adults

**DOI:** 10.3758/s13423-026-02880-0

**Published:** 2026-04-14

**Authors:** Leigh B. Fernandez, Muzna Shehzad, Lauren V. Hadley

**Affiliations:** 1 Center for Cognitive Science, Psycholinguistics Group, University of Kaiserslautern-Landau, Postfach 3049, 67653 Kaiserslautern, Germany; 2 Hearing Sciences - Scottish Section, School of Medicine, University of Nottingham, Leve1 3 New Lister Building, Glasgow Royal Infirmary, 16 Alexandra Parade, Glasgow, G31 2ER UK

**Keywords:** Prediction, Cognitive load, Executive resources, Hearing loss

## Abstract

Whether linguistic prediction requires executive resources is currently unclear. Supportive evidence comes from reduced/delayed prediction in populations with fewer available executive resources, such as children or second-language speakers. However, it is possible that differences in these groups instead stem from differences in linguistic experience. In this study we test prediction using the visual world paradigm, investigating two factors proposed to reduce the availability of executive resources during listening: memory load and hearing loss. Specifically, we apply a visuo-spatial cognitive load manipulation in older adult groups matched in linguistic experience and working memory capacity, but differing in hearing ability. Without a load task, participants with normal hearing were earlier to look to the predictable target relative to those with hearing loss, suggesting a cognitive cost to hearing loss that impacts prediction mechanisms. With a load task, participants with normal hearing were later to look to the predictable target relative to the no-load task, while participants with hearing loss showed no difference by load. Our findings suggest that taxing executive resources via load delays prediction specifically in older adults with normal hearing. While the lack of load effect in the hearing loss group is surprising, it could reflect greater resilience to challenge, altered prediction strategies, or differences in task prioritization. All reported patterns were specific to predictable items and were not seen in neutral items. Importantly, differences between groups cannot be explained by differences in linguistic experience, but rather support findings that executive resources play an important role in prediction.

**Supplementary Information:**

The online version contains supplementary material available at 10.3758/s13423-026-02880-0.

Language processing involves complex mechanisms, yet we are easily able to follow speech, even under challenging conditions (e.g., with noise; whilst engaging in additional activities such as driving). This ability is supported by listeners making predictions of what is likely to come next, which helps increase accuracy (less stimulus information is required to identify a predicted word), increase processing speed (predicted words are more easily identified and accessed), and decrease cognitive cost (the resources required for identification and access are reduced; e.g., Holt et al., 2021). Over the past 30 years or so, research investigating prediction in college-aged first language (L1) speakers has established that individuals are able to use multiple sources of information to make linguistic predictions during speech listening (for reviews, see Ferreira & Chantavarin, 2018; Huettig, 2015), with findings being extended to different populations, including children (e.g., Gambi et al., 2018), second language (L2) speakers (e.g., Kaan & Grüter, 2021), and older adults (e.g., Payne & Silcox, 2019). Yet there are several aspects of prediction that are not well understood.

One contentious question regarding prediction is whether it is a necessary part of language processing. While some current frameworks argue that this is indeed the case (e.g., Fitz & Chang, 2019; Rabovsky et al., 2018), others argue that while prediction is useful, it is by no means a requirement (e.g., Huettig & Mani, 2016; Pickering & Gambi, 2018). A key distinction between these views stems from the hypothesized relation between prediction and executive resources (Ryskin et al., 2020)—executive resources being the high-level cognitive processes that aid in the regulation of goal-directed behavior. Executive resources include abilities such as inhibiting irrelevant information as well as maintaining, manipulating, and updating information in working memory (Friedman & Miyake, 2017). Critically, those arguing that prediction is a necessary part of language processing do not propose executive resource involvement (e.g., Fitz & Chang, 2019; Rabovsky et al., 2018), whereas those arguing that prediction is optional do propose executive resource involvement (e.g., Huettig & Mani, 2016; Pickering & Gambi, 2018). Evidence of prediction being dependent on executive resources predominantly comes from studies manipulating resource availability or studies comparing populations.

The authors are aware of only four studies that have directly manipulated resource availability within participants during an explicit assessment of prediction (i.e., using a paradigm that affords measurement of target activation before bottom-up confirmation; Allison et al., 2025; Harel-Arbeli et al., 2023, 2025; Ito et al., 2018). These studies use the visual world paradigm (VWP), which is a key method for assessing prediction that involves participants viewing an array of images while hearing spoken sentences that mention some (or all) of the images. The time course of participants’ looks to the images provides insight into prediction and integration of linguistic information during listening, with prediction being inferred if looks to the target increase prior to the target being spoken (e.g., Allopenna et al., 1998; Tanenhaus et al., 1995). Prediction in the VWP can stem from multiple sources, such as the properties of the agent or verb or semantic context. Using this paradigm, Ito et al. (2018) tested L1 and L2 speakers of English listening to sentences where the verb either constrained the upcoming object (predictable sentences) or did not (neutral sentences). Half of the participants listened with no load, and half listened while engaging in a cognitive load task (which involved remembering a list of five words throughout the VWP trial). They found that prediction in the predictable sentences was delayed in the load task relative to the no-load task, with both L1 and L2 speakers being similarly affected. As there was no such effect in the neutral items, this suggests that load specifically impacted prediction processes. Allison et al. (2025) also tested L2 speakers of English, this time listening to sentences where the agent and verb combinedly constrained the upcoming object (predictable sentences) or did not (neutral sentences). In this study, load was manipulated with a visuo-spatial task (i.e., Corsi block tapping task; Corsi & Michael, 1972). As well as a no-load condition, participants engaged in a low-load condition (remembering two locations in a grid of nine) and a high-load condition (remembering four locations in a grid of nine). After each VWP trial, participants were asked to recall the locations in the correct sequence. Allison et al. found a reduction in predictive eye movements as load increased, with the reduction being specific to the predictable rather than neutral items. Finally, across two studies that analyzed the same visual world dataset—one investigating eye movements (Harel-Arbeli et al., 2023) and the other investing pupil dilation (Harel-Arbeli et al., 2025)—the authors tested younger and older L1 speakers listening to sentences in which the semantic context constrained the target (predictable sentences with or without a semantic competitor) or did not (neutral sentences). They presented stimuli under low load (remembering one digit) or high load (remembering four digits) and found a reduction in looks to the target in the high- relative to low-load conditions, but only in the predictable sentences. Older adults were more affected by load than younger adults. As all of these studies found that increased load led to reduced or delayed prediction, they suggest that executive resources are required to make predictions.

In terms of population comparisons, reduced prediction has been reported in several populations with fewer available resources. For example, research has found that children with lower working memory and lower language proficiency are less likely to predict than children with higher working memory and proficiency (Mani & Huettig, 2012) and when demands on executive resources are high (Maquate & Knoeferle, 2021). These findings may in part be due to children having a less developed prefrontal cortex which is crucial for cognitive control (Thompson-Schill et al., 2009). Similarly, decreased efficiency in prediction for older adults is associated with decreased working memory capacity and inhibitory skills (e.g., Harel-Arbeli et al., 2023; Huettig & Janse, 2016; Huettig & Mani, 2016) which may be the result of age-related cognitive declines in executive functions (e.g., Nyberg et al., 2012; Pliatsikas et al., 2018).

Additionally, L2 speakers show reduced prediction efficiency which has been attributed to the increased executive resource demands that come with processing a second language, leaving less capacity for prediction during L2 processing (Ito & Pickering, 2021; Kaan, 2014; Ribu et al., 2024; Segalowitz, 2016).

While together this work could suggest the involvement of executive resources in prediction, confounding differences between groups may contribute to these findings. For example, children’s limited experience may lead to weaker links between lexical and semantic concepts, making it harder for them to make predictions (Mani & Huettig, 2014). Additionally, older adults have more cumulative linguistic experience compared with younger L1 speakers, which may lead to predictions that do not align with younger speakers (on whom many stimulus sets have been normed). Thus, differences between such groups could instead be an artefact of the erroneous assumption that the outcome of predictions should be the same across populations with differing experience (Ryskin et al., 2020), highlighting the importance of either matching linguistic experience between groups, or generating stimuli specific to each group in question.

To extend such prior work, we combine these two resource manipulation and population comparison approaches, focusing on older adults with varying hearing abilities. Specifically, we address age-related (i.e., postlingual) hearing loss, because in spite of similar linguistic experience to those with normal hearing, degraded auditory signals lead to a greater reliance on the cognitive system to extract meaning (e.g., Peelle, 2018; Pichora-Fuller et al., 2016). Such increased reliance increases cognitive demands (Rönnberg et al., 2013), which is a key contributor to listening effort (Peelle, 2018). Indeed listening effort has been reported to increase with hearing loss (e.g., McLaughlin et al., 2021; Ohlenforst et al., 2017; Pichora-Fuller et al., 2016) depleting the executive resources available for language processing and reducing the pool available for generating predictions (Fernandez et al., 2024). To date, the only study directly testing prediction in people with hearing loss (Fernandez et al., 2025a) found that prediction specifically was delayed for people with hearing loss when listening demands were increased by stimuli that were intelligible but effortful to listen to (with no such effects in neutral sentences). Thus, older adults with varying hearing abilities are ideal to address questions regarding executive resource availability while holding linguistic experience constant.

## Current study

In sum, the role of executive resources in prediction is not well understood, but several strands of research suggest that executive resources play an important role in prediction. However, it is possible that the prediction differences reported for many of the groups assumed to differ in executive resource availability rather stem from differences in linguistic experience. In the current study, we investigate how prediction is impacted by two factors affecting the availability of executive resources during listening: memory load and hearing loss. Specifically, we test older adults with and without hearing loss, using items specifically normed for predictability with older adults. If prediction relies on executive resources, we hypothesize that people with hearing loss (PwHL) should show delayed prediction relative to people with normal hearing (PwNH) when listening at the same acoustic presentation level (70 dB), given that the former group experience greater demand and thus has to invest more listening effort, leaving fewer available resources to allocate to prediction. Given that the presentation level in this experiment (70 dB) is nonetheless intelligible we do not expect this difference in the timing of looks to the target in the neutral items because PwHL seem to integrate lexical information similarly to PwNH (Fernandez et al., 2025a). In addition, using the Corsi block task, we manipulate cognitive load within participants to test whether an additional visuo-spatial working memory task influences prediction at an individual level. By doing this within each group we can assess how prediction delays scale with executive resource depletion. We hypothesize that load will delay prediction, and expect that this delay may be greater for PwHL due to the accumulation of difficulty in the load condition.

## Methods

The sample size for this study was based on a recent simulation-based power recommendation for VWP studies (Prystauka et al., 2024). Prystauka and colleagues (2024) showed that verb-based prediction effects of the magnitude typically reported can be reliably detected with 20–30 participants and 12–16 items per condition, achieving approximately 80% power. Our study meets these recommendations (*N* = 30/41 participants per group; 18 items per condition), thus providing sufficient statistical power to detect differences.

### Participants

This study included 71 older adults, who were categorized into two distinct subgroups—30 PwNH, defined as having a worse ear pure tone average (WEPTA), ranging from −10 to 30 dB, and 41 PwHL, defined as having a better ear pure tone average (BEPTA), ranging from 30 to 65 dB (i.e., the worse ear of the PwNH was better than the better ear of the PwHL). The average BEPTA for PwNH was 17.96 dB, and the BEPTA for PwHL was 50.40 (see Table 1). According to the World Health Organization (2021), the normal hearing range from 0 to 20 dB in BETPA, mild hearing loss is between 20 to less than 35 dB, and moderate hearing loss is 35 to less than 50 dB. Thirty-seven of 41 PwHL had a sloping audiogram consistent with age-related sensorineural hearing loss, and 36 were hearing-aid users. If participants used hearing aids, they were asked to remove them for the hearing test and during the study. All participants had normal or corrected-to-normal vision. An additional eight individuals were disqualified because their hearing did not qualify for either subgroup, and 13 individuals (seven PwHL/six PwNH) due to atypical eye-movement strategies during the study (i.e., reporting in the postexperiment questionnaire that they did not look at the pictures at all, or used their eyes to recreate the Corsi pattern). This research study received ethical approval from the University of Nottingham Faculty of Medicine and Health Science Research Ethics Committee (REC reference: FMHS 423–1221). All participants provided voluntary consent and received £20 in compensation for their time (see Table 1 for additional participant information).

**Table 1:** Participant information

	PwNH	PwHL
*N* (m/f)	30 (13/17)	41 (21/20)
Age (*sd*)	65.63 (7.30)	71.27 (6.43)
Better ear average dBHL (*sd*)	17.96 (6.75)	50.40 (9.88)
Worse ear average dBHL (*sd*)	22.00 (15.26)	55.72 (11.89)
SicSpan WM score (*sd*)	21.11 (5.69)	18.56 (5.81)
SicSpan intrusion score (*sd*)	2.85 (2.07)	3.12 (1.90)

Importantly, participants did not significantly differ in scores of working memory or intrusion (an inhibition metric), as measured via the SicSpan. While PwNH were approximately 5 years younger than PwHL, this slight age difference did not therefore appear to impact executive functioning, and groups were considered matched in executive resource capacity. For all group level comparisons see the Supplementary Materials (S1) and OSF (https://osf.io/48whs) for complete data and analyses.

### Design

The experiment included two counterbalanced blocks, one without a load task and one with a concurrent visuo-spatial load task. Each block began with three practice trials, and included 52 stimuli: 27 critical items (nine constraining expected, nine constraining unexpected, nine neutral), as well as 22 fillers. Therefore each participant encountered 27 critical items under load and 27 critical items under no load (total = 54 critical items).

### Stimuli

The sentence stimuli were adapted from Fernandez et al. (2025b) and were predictability normed with L1 UK-English older adults (age approximately 68 years; see Fernandez et al., 2025b, for details). These sentences all followed the same structure (see Table 2): The [agent] [verb] the [critical word].

**Table 2:**
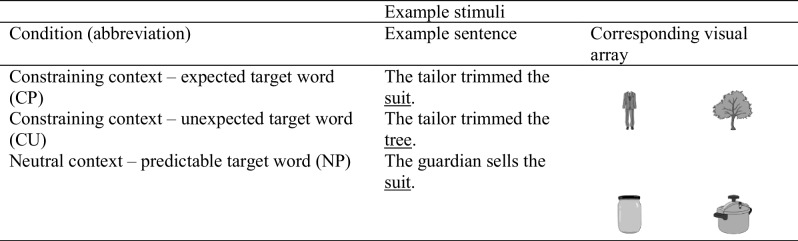
Example stimuli

The critical sentences were created in trios^1^ (see Table 2). Each trio consisted of a constraining context with a predictable target word (CP), the same constraining context with an unexpected target word (CU), and a neutral context (which was plausible but not predictable) ending with the same target word used in the predictable condition (NP). Each participant saw 54 critical trials: 18 CP, 18 CU, and 18 NP. The CP and CU items were counterbalanced, so that no participant encountered the same sentence context twice. However, as the CU and NP versions of each item shared no linguistic overlap, participants additionally saw the NP version corresponding to each of their CU items (i.e., if they saw *The tailor trims the tree* they would additionally see *The guardian sells the suit*, but they would not see *The tailor trimmed the suit*.) The CU and NP forms of a given item shared the same visual display (as noted below the image location was randomized) and were presented in different counterbalanced blocks within the experiment. 

Each sentence trio had a corresponding visual comprised of four images taken from the British English MultiPic databank (Duñabeitia et al., 2018), which were 300 × 300 pixel greyscale jpegs differing in initial phonemes. Table 2 provides an example sentence trio. The two distractors were plausible NP objects but not plausible CP or CU objects. The images were counterbalanced across items so each image type occurred at each location in the array 25% of the time. The image locations were the same for CP and NP items (but participants did not see both version of the CP and NP item), and the CU images shared images with the NP item but location was rotated (to ensure participants did not map object locations).

### Auditory information

The items were recorded by a male L1 speaker of Scottish English at a 48000 Hz sampling rate using Audacity recording software (Audacity Team, 2001). The items were normalized post hoc such that each corresponding word across items was the same length, thus all items were exactly the same duration. Stimuli were presented at 70 dB (see Fernandez et al., 2025b, for additional information).

### Apparatus

The VWP experiment was run in a sound-attenuated booth. Participants were positioned using a chin rest approximately 80 cm from a 17-in. ADI MicroScan A715 (Model Q17) monitor, with a resolution of 1,024 × 768 and a refresh rate of 60 Hz. During the experiment, their right eye was recorded using a desktop-mounted EyeLink 1000 Plus with a sampling rate of 1000 Hz, while auditory stimuli were presented through over-ear AKG reference headphones (7202). Headphone checks were performed daily by the experimenter, and calibration was performed using a sound level meter.

### Procedure

The procedure was identical for PwHL and PwNH. Participants first underwent audiometric testing to assess their four-frequency pure tone average. In the main experiment participants heard spoken sentences (presented at 70 dB) and saw an array of four images, while their eyes were tracked. Their task was to listen to the sentence and then click the image from the visual array that best matched the sentence they just heard. They were informed that in one block they would see an array of squares before the sentence that would light up in sequence. For those trials their secondary task was to remember the sequence and report it back afterwards.

The VWP task began with a standard 9-point calibration, and all trials began with drift correction. Each trial was divided into the encoding phase, the VWP phrase, and the recall phase. The encoding phase began with nine randomly presented squares being presented on a grey background for 750 ms. In the no-load condition, the squares then remained on-screen for an additional 2,250 ms with no additional task, whereas in the load condition, three random squares sequentially changed blue for 750 ms each, and participants were tasked with remembering the sequence of blocks that changed color. The VWP phase then began with a fixation cross presented in the center of the screen for 1,000 ms, after which an array of images was then displayed for 2,000 ms. At this point, the auditory stimulus was presented. After the auditory stimuli ended, the images remained on the screen for an additional 2,000 ms, at which point a border encompassed the array and the mouse icon appeared, indicating they could click on one of the images. Finally, the recall phase involved presentation of the same block sequence from the encoding phase. In the no-load condition, the squares remained on the screen for 1,500 ms with no additional task, whereas in the load condition, participants were instructed to click on the squares that had changed color in the encoding phase, in order, with no time limit. The eye-tracking task was self-paced, and participants could take breaks as needed (between trials with subsequent recalibration). After each block, participants completed the NASA Task Load Index (TLX; Hart & Staveland, 1988) to provide a subjective score of the effort required for the block they just completed. The effort rating used a 0–100 scale, with 0 being *very low effort* and 100 being *very high effort*. After the load block specifically, they were also asked “What strategies did you use to remember the squares, if any?”. Following the eye-tracking task, participants performed a complex working memory task (Size-Comparison Span [SicSpan]; Sörqvist et al., 2010), were debriefed, and were compensated.

### Analysis

#### Behavior

Comprehension accuracy, defined as the number of trials in which the participant clicked on the stimulus target was 97%, and incorrectly answered trials were removed (0.2% of items were removed for PwNH and 4.8% for PwHL). See Table 3 for mean accuracy by group, load, and stimulus type, and see the Supplementary Materials (S2) for more detail and statistical comparisons.

**Table 3:** Mean comprehension accuracy by task by group, load, and stimulus type

Mean (*sd*) comprehension accuracy (%)				
	PwNH		PwHL	
Type	No load	Load	No load	Load
CP	100 (0)	100 (0)	98.37 (12.66)	99.19 (8.99)
CU	98.89 (10.5)	100 (0)	89.7 (30.43)	90.51 (29.34)
NP	100 (0)	100 (0)	96.75 (17.76)	96.48 (18.46)

Effort, assessed using a questionnaire at the end of each block, was significantly different between load with 36.05% in the no-load condition and 74.36% in the load condition, and was significantly different between groups at 50.85% for PwNH and 58.40% for PwHL. An interaction suggested a greater load effect in PwNH than PwHL, but individual comparisons failed to find this effect. See Table 4 for mean accuracy by group and load, and see the Supplementary Materials (S1) for more detail and statistical comparisons.

**Table 4:** Mean effort score task by group and load

PwNH		PwHL	
No load	Load	No load	Load
27.53 (26.44)	74.17 (23.30)	42.29 (31.56)	74.51 (23.76)

Complementing this, accuracy on the Corsi task, taking into account both sequence and order, was significantly different between groups at 52% for PwNH and 36% for PwHL. See Table 5 for mean accuracy by group, and see the Supplementary Materials (S1) for more detail and statistical comparisons.

**Table 5:** Mean Corsi accuracy by group

Mean (*sd*) Corsi accuracy (%)		
Type	PwNH	PwHL
CP	55.93 (49.74)	37.4 (48.45)
CU	44.44 (49.78)	35.23 (47.83)
NP	54.44 (49.89)	35.77 (48)

#### Prediction timing

Before estimating divergence timing, looks to the images were tested to ensure there was a difference between the target and the distractor image fixation trajectories. A generalized additive mixed model (GAMM) was fit to model both the linear and nonlinear relationships (Wood, 2017) that are inherent in VWP data. For each comparison, the main dependent variable was the proportion of fixations to the target and distractor images over time. The model included fixed effects of image (target vs. distractor), load (no load vs. load), and group (PwNH vs. PwHL), and their interactions. Condition-specific smooths over time, and random smooths for participant and items over time were included to account for individual and item-wise variability in the pattern of looks to the images (see OSF).

To test the timing of looks to the images across conditions and group, we used divergence point analysis (DPA; Stone et al., 2021). The DPA is a nonparametric analysis that uses bootstrapping to estimate the time at which looks between two images diverge. The timing is determined by finding the earliest point in which looks between two images significantly differ (using *t* tests) for at least 200 ms (i.e., 10 consecutive 20-ms bins). Two thousand new datasets are then generated using bootstrapping sampling across participant, time, and image, after which the mean divergence time and a 95% confidence interval can be estimated. CIs that contain 0 do not support a reliable difference, while those that do not contain 0 do support a reliable difference. This analysis allows us to compare effect latencies across groups while dealing with the nonindependence of fixations and decreasing the likelihood of Type I errors (Stone et al., 2021).

We ran two separate DPA analyses.^2^ The first DPA investigated prediction. The CP and CU items were equivalent in structure and contextual constraint until the target word (e.g., in the example in Table 2 they both include *The tailor trimmed the*), hence we collapsed these items to compare looks to the predictable CP image (*suit*) versus the CU image (*tree)* from the onset of the sentence to the offset of the article prior to the target (i.e., the time span that afforded prediction of the CP image in both version of the stimuli). Thus the prediction analysis comprised 18 critical items in each load condition. This aggregation of CP and CU stimuli was done within participants, yielding mean fixation proportions to each image during the time span allowing prediction under each load condition. Note that although each participant saw only one contextually constraining version (CP or CU) of a given item, the linguistic input preceding the target for the CP and CU versions of an item was equivalent, justifying the combined analysis.

The second DPA investigated lexical integration. In the NP items (*The guardian sell the suit*), we compared looks to the CP image (*suit*) versus CU image (*tree*) from the onset of the sentence out 2,500 ms (the target occurs 1,440 ms). The lexical integration analysis comprised nine critical items in each load condition. Due to the 2 × 2 design (PwNH/PwHL, no load/load), each DPA compared the timings of: (1) PwNH–no load versus PwHL–no load, (2) PwNH–load versus PwHL–load, (3) PwNH–no load versus PwNH–load, and (4) PwHL–no load versus PwHL–load. Differences in divergence times between the groups and conditions were computed from the same bootstrap distribution and are reported in Table 6. All analyses were run using R (R Core Team, 2018); see OSF (https://osf.io/48whs) for data, analysis, code, and information on all R packages and versions.

**Table 6:** Difference in divergence times [95% confidence intervals] across load and group

Comparison (sentence type)	Image comparison	PwNH– load vs PwNH–no load	PwHL–load vs PwHL–no load	PwHL–no load vs PwNH–no load	PwHL– load vs PwNH–load
Prediction (CP & CU)	Suit vs tree	**263.38 [180, 340]** **No load: 705** **Load: 968**	−47.34 [−180, 120] No load: 829 Load: 782	**123.99 [20, 220]** **PwNH: 705** **PwHL: 829**	−183.44 [−280,10] PwNH: 968 PwHL: 782
Lexical integration (NP)	Suit vs tree	62.75 [−20, 140]	−63.87 [−120, 0]	77.6 [0, 160]	−49.02 [−100, 20]

If the CI contains 0, the comparison is not considered reliably different. If the CI does not contain 0, the comparisons is considered reliably different

## Results

**Fig. 1:**
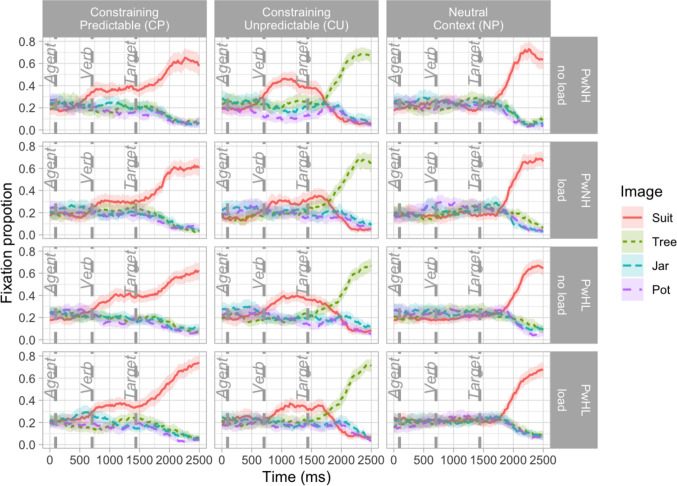
Mean proportion of looks across to correctly answered items by sentence type, participant group, and load. (Color figure online)

Figure 1 shows the fixation proportion to the four images in the visual array over time. Table 6 provides the difference in divergence times (with 95% confidence intervals) across load and group.

In both of our two analyses, we first established that looks to the objects differed across time, with GAMMs revealing main effects of Image and significant higher-order interactions (all *p* values < .001), and all condition-specific smooths being significant (all *p* values < .001). This confirmed that looks to the target and distractor diverged reliably over time (see OSF for analyses). Having established that these differences existed, we used a DPA to determine when divergences occurred.

**Fig. 2:**
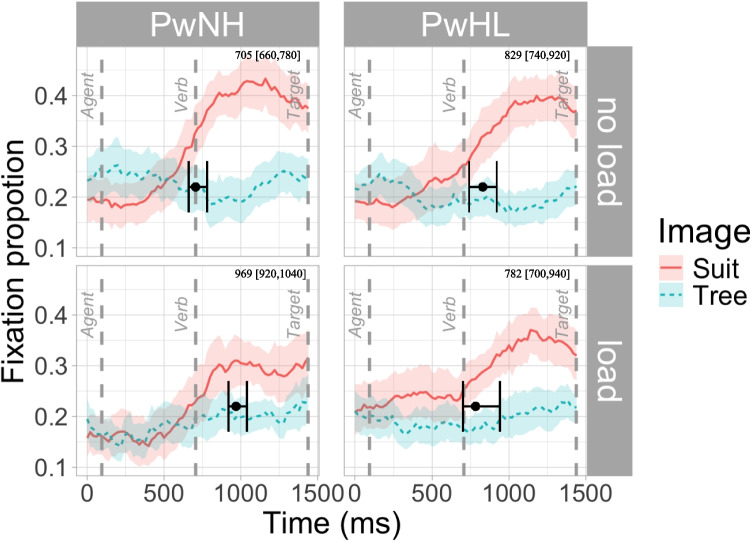
Divergence point and 95% confidence intervals superimposed on the fixation proportion of looks to the CP target and the CU target in CP items across load and group. Divergence times [95% confidence intervals] indicated in top right corner. (Color figure online)

The first DPA collapsed across the CP and CU items and compared looks to the predictable CP image (*suit*) versus the CU image (*tree*). Divergence timing in PwNH was later in the load relative to the no-load condition (263.38 ms [CI: 180, 340]); given the CI does not contain 0, this supports a reliable difference between conditions. In PwHL there was no reliable difference between load conditions. In the no-load condition, the PwHL had a reliably later divergence time relative to the PwNH (123.99 [20, 220]), but there was no reliable difference between groups in the load condition (−183.44 [−280, 10]). See Fig. 2 and Table 6.

**Fig. 3:**
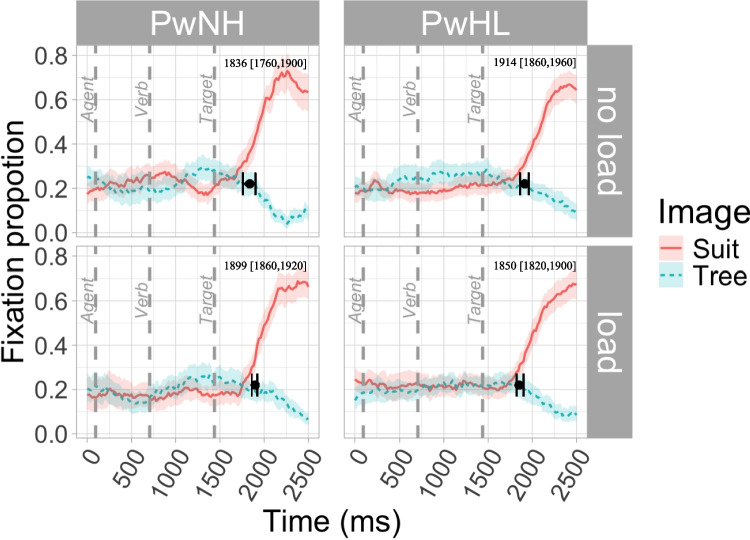
Divergence point and 95% confidence intervals superimposed on the fixation proportion of looks to the CP target and the CU target in NP items across load and group. Divergence times [95% confidence intervals] indicated in top right corner. (Color figure online)

The second DPA focused on the NP items and compared looks to the NP image (*suit*) versus CU image (*tree*). As all CIs include 0, they do not support reliable differences between groups or conditions. While the comparison between PwNH and PwHL in the no-load condition (77.6 ms [0, 160]) and the comparison between PwHL in the load versus no-load condition both seem to be approaching a difference (−63.87 ms [−120, 0]), given that the CI includes 0, we do not consider this difference reliable (see Fig. 3 and Table 6).

## Discussion

While evidence is relatively robust that individuals actively make predictions during language processing (e.g., Huettig, 2015) it remains unclear whether prediction requires investment of executive resources (Ryskin et al., 2020). While participant groups with fewer available executive resources often show reduced or delayed prediction, it is possible that these prediction differences rather arise from differences in linguistic experience (Ryskin et al., 2020). To investigate prediction in groups matched in experience but differing in executive resource availability, we therefore tested older adults with and without hearing loss using items normed with an older population. In addition, we manipulated cognitive load using a visuo-spatial working memory task previously shown to decrease prediction with second language speakers (Allison et al., 2025). We hypothesized that PwHL would show delayed prediction relative to PwNH, but the two groups would not differ in the timing of looks to the target in the neutral items, given that they do not require prediction. Additionally, we hypothesized that the load task would delay prediction relative to having the no load task, and this delay may be greater for PwHL due to the accumulation of difficulty.

We found that in the no-load condition, PwNH were reliably earlier to make predictions than PwHL. While Fernandez et al. (2025a) only found differences in prediction timing between PwNH and PwHL for stimuli more complex than those used here, it is possible that combining the load conditions may have impacted performance due to fatigue or strategy development. When it came to the addition of the load task, this impacted PwNH by delaying predictions by approximately 250 ms, but strikingly had no impact on PwHL. Indeed in the load condition, prediction timings were no longer reliably different between PwHL and PwNH. This suggests that taxing working memory in PwNH causes a delay similar to that caused by the additional demands of hearing loss, and that a shared pool of resources may underlie the mapping between (degraded) speech input and mental representations, processing visual displays, maintaining visuo-spatial sequences, and generating linguistic predictions. When looking at language processing that does not involve prediction (i.e., the NP items), we no longer see any reliable differences between groups and/or load. It is important to acknowledge that for neutral items, the comparison between (1) groups in the no-load condition and (2) load conditions in PwHL were approaching a difference (both have 0 as a CI). While these differences are not reliable, the former may indicate that hearing loss causes a general delay in processing, though we specify that when processing neutral sentences with clear speech, the delays are minimal for PwHL (i.e., 77 ms), but when predicting, those delays increase and reach significance (causing the significant 124ms difference). The latter, potentially suggesting that PwHL are quicker to integrate heard words in higher load, is unexpected. We note again that the CIs include 0 and thus cannot be used to generate conclusions but raise the possibility that this could relate to our suggestion below that PwHL are more resilient to challenge, and thus it is possible that they show some form of benefit. Together, our findings suggest that both hearing loss and cognitive load lead to specific delays in prediction, and thus that taxing executive resources (either through more demanding listening or the inclusion of an additional task) impacts prediction mechanisms.

Interestingly, however, we did not find an effect of load for PwHL. While unexpected, we propose that this is likely the result of PwHL using a different task strategy to PwNH: Specifically, PwHL may have chosen to prioritize the language processing part of the task rather than investing their limited resources into the memory load part of the task. This is supported by PwHL showing lower accuracy on the Corsi task than PwNH (36% as opposed to 56%; see S2), in spite of groups having similar working memory capacity as assessed via SicSpan. Alternatively, PwHL may be more resilient to cognitively demanding tasks, with their greater experience in demanding scenarios leading to the development of compensation strategies. Indeed PwHL may rely more on automatic predictions than nuanced and tailored predictions (Corps et al., 2022; Fernandez, et al., 2025c; Pickering & Gambi, 2018) given that they are less costly, thus allowing prediction even under cognitive demanding scenarios.

It is worth noting that the PwHL and PwNH were all recruited from the same database and geographic area and while we do not anticipate systematic differences between the groups in terms of education, multilingualism, work status, and so on, we did not collect detailed background measures. To further dig into differences in performance between groups, systematically investigating these background measures may offer valuable insight into how such factors impact prediction processes across groups of speakers.

Overall, these findings present an interesting picture, suggesting that executive resource availability is critical for prediction. This has implications for theories of prediction, given that the group differences cannot be explained by differences in linguistic experiences (e.g., L1 vs. L2) or norming procedures (e.g., items normed by young adults but tested with older adults). Our findings that prediction is sensitive to the availability of executive resources suggests that prediction cannot be an automatic obligatory aspect of language processing. Obligatory processes would operate regardless of resource availability, yet the evidence presented here suggests that prediction is modulated by resource availability.

However, the question remains, what prediction processes specifically require the availability of executive resources? The theory of *prediction-by-production* (Pickering & Gambi, 2018; Pickering & Garrod, 2007) proposes that prediction occurs via the production system, with listeners covertly imitating the motor commands of the speaker and using forward models to predict upcoming input. Language production is largely believed to be nonautomatic and involve several steps which require executive resources (e.g., Bock, 1982; Hartsuiker & Barkhuysen, 2006), so it is possible that reduced cognitive resources impact the prediction-by-production stage. Alternatively, it is also possible that executive resources are required by the quirks of the visual world paradigm, rather than by language processing in itself. Huettig and Mani (2016) argue that during a VWP task, comprehenders must process the visual array and create linguistic and conceptual representations, match these to the spoken input, then map these back to the location of the images on screen via working memory. Thus, executive resources could be necessary for integrating the displayed items with the heard speech, leading to delays when under load. Furthermore, individuals with hearing loss may rely more heavily on visual information (Peelle & Wingfield, 2016), which may increase the executive resources required for mapping and integrating the visual display as the speech information unfolds. More work would be required to investigate the aspects of working memory required for prediction, and how the paradigm itself draws on executive resources.

## Conclusions

We tested how the availability of executive resources affected prediction by manipulating resource availability via a load task in a study of older adults with and without hearing loss. We found that in the no load condition, PwHL were delayed in making predictions relative to PwNH. This highlights the cognitive costs of hearing loss and its impact on prediction processes specifically. Furthermore, we found that the addition of the load task delayed prediction in PwNH but not in PwHL. Thus taxing executive resources in PwNH leads to the hypothesized delay in prediction, but PwHL behave differently. We interpret this lack of load effect in PwHL as the consequence of prioritization of the linguistic task over the load task, greater resiliency to more demanding tasks based on prior experience, or a greater reliance on more automatic prediction. Given that these findings only occurred in the predictable items that were normed with older adults, they cannot be explained by experience differences, but rather support executive resource availability being critical for linguistic prediction.

## Supplementary Information

Below is the link to the electronic supplementary material.

Supplementary file1 (DOCX 20 KB)

Supplementary file2 (DOCX 94 KB)

Supplementary file3 (DOCX 248 KB)

## Data Availability

The data for this experiment are available online (https://osf.io/48whs). Materials were taken from Fernandez et al. (2025b), and none of the experiment was preregistered.

## Footnotes

1 We took a subset of 54 sentence trios from the 72 used in Fernandez et al. (2025b); the remaining 18 sentence trios were adapted as fillers which serve as the basis of another study.

2 See the Supplementary Materials (S3) for a DPA analysis on the CU items alone.

## Code availability

The code for the analyses is available online (https://osf.io/48whs),

## Declarations

### Open practices statement

The data and code for this experiment are available online (https://osf.io/48whs), and this experiment was not preregistered.
