# Supplementary material for: Involvement of executive resources in prediction: Effects of load and hearing loss in older adults
Source: Psychon Bull Rev. 2026 Apr 14;33(4):135. doi: 10.3758/s13423-026-02880-0 (PMC13079493; doi:10.3758/s13423-026-02880-0)
Supplement: Supplementary file 1 — Supplementary file1 (DOCX 20 KB) [file 13423_2026_2880_MOESM1_ESM.docx]

Group level comparisons

To test differences between groups we ran t-tests to compare age, better ear average, and SicSpan WM scores. see Table S1.1.

Table S1.1. Participant information

|  | PwNH | PwHL |
| --- | --- | --- |
| N (m/f) | 30 (13/17) | 41 (21/20) |
| Age (sd) | 65.63 (7.30)  PwNH-PwHL *p*=<0.01 ***** | 71.27 (6.43) |
| Better ear average dBHL (sd) | 17.96 (6.75)  PwNH-PwHL *p*=<0.01 ***** | 50.40 (9.88) |
| Effort rating | Load – 74.17 (23.30)  No Load – 27.53 (26.44) | Load – 74.51 (23.76)  No Load – 42.29 (31.56) |
|  | No load-Load *p*=<0.01 *****  PwNH-PwHL *p*=<0.05 *****  Load*Group *p*=<0.05 ***** (however, pairwise comparisons showed no differences between load by group) | |
| SicSpan WM score (sd) | 21.11 (5.69)  *No differences* | 18.56 (5.81) |
| SicSpan intrusion score (sd) | 2.85 (2.07)  *No differences* | 3.12 (1.90) |

We found that PwNH were younger approximately 5 years younger (*t*(58)=-3.37, *p*<0.01) and had a better ear average (*t*(69)=-16.43, *p*<0.01) than PwHL, however there were no differences in WM^[[1]](#footnote-1)^ (*t*(54)=-54, *p*=0.10) or intrusion score (*t*(51)=-0.53, *p*=0.60), suggesting that even though the groups differed slightly in age this did not impact their WM capacity. Additionally, we tested effort ratings recorded during the different parts of the experiment using a linear mixed effects model with the fixed effect of group (PwNH/PwHL; sum contrast coded (.5/-.5) with PwNH serving as the baseline) and load (No load/Load; sum contrast coded (.5/-.5) with No load serving as the baseline), and their interaction. Because participants provided repeated measures (one score for No load and one score of Load) we included the random effect of participant. The model revealed that ratings were higher in the Load relative to the No load condition (est=46.63, *t* =8.88, *p*<0.01), PwHL had higher effort ratings than PwNH (est=14.76, *t* =2.30, *p*<0.05), and there was an interaction (est=-14.41, *t* =-2.09, *p*<0.05). Post-hoc comparisons using the Tukey HSD test revealed that effort ratings were higher in the Load relevant to the No load condition for both PwNH (*p*<0.01) and PwHL (*p*<0.01), however, the comparisons between PwNH Load vs PwHL Load and PwNH No load vs PwHL No load did not reach significance (*p’s*>0.09). See Table S1.2 for model output and means.

Table S1.2. Effort model output ad means

| Predictor | | Estimate | *SE* | *df* | *t* | | *p* | |
| --- | --- | --- | --- | --- | --- | --- | --- | --- |
| **Intercept** | | **27.53** | **4.88** | **117.27** | **5.64** | | **< .001***** | |
| **Load (Load)** | | **46.63** | **5.25** | **69.00** | **8.88** | | **< .001***** | |
| **Group** | | **14.76** | **6.42** | **117.27** | **2.30** | | **.023*** | |
| **Load (Load) × Group** | | **-14.41** | **6.91** | **69.00** | **-2.09** | | **.041*** | |
| **Mean effort (SD)** | |  |  |  | |  | |  |
| Load | PwNH | PwHL |  |  | |  | |  |
| No load | 27.53 (26.44) | 42.29 (31.59) | |  | |  | |  |
| Load | 74.17 (23.31) | 74.51 (23.76) |  |  | |  | |  |

See OSF for data and analyses.

1. Note: 4 PwHL and 9 PwNH did not complete the SicSpan task, therefore the working memory analyses contains 37 (PwHL)/21 (PwNH) [↑](#footnote-ref-1)
