# Supplementary material for: Involvement of executive resources in prediction: Effects of load and hearing loss in older adults
Source: Psychon Bull Rev. 2026 Apr 14;33(4):135. doi: 10.3758/s13423-026-02880-0 (PMC13079493; doi:10.3758/s13423-026-02880-0)
Supplement: Supplementary file 2 — Supplementary file2 (DOCX 94 KB) [file 13423_2026_2880_MOESM2_ESM.docx]

**Accuracy – analysis, results, and discussion**

Two models were run to test accuracy: the first investigated the accuracy on the sentence comprehension task and the second investigated the accuracy on the Cosi task. For the sentence comprehension task we analyzed the accuracy of clicking on the correct image (i.e., the image that matched the sentence). For the Corsi task we analyzed accuracy in the recall phase (both in terms of location and order), and therefore this analysis only included data from the load condition.

For both models, accuracy was coded as 1 (correct)/0 (incorrect) and submitted to a logit mixed effects model. The fixed effects were type (CP/CU/NP) which was treatment coded (with CP as the baseline) and group (PwNH/PwHL) which was sum contrast coded (.5/-.5). For the comprehension accuracy model, there was additionally a fixed effect of load (load/no load) which was sum contrast coded (.5/-.5). The random effects of item and participant were maximally specified (Barr et al., 2013). If the maximal model did not converge the random effects structure was simplified (as described in the results section). An omnibus test was used to test for main effects and interactions, and contrasts were only interpreted if the comparison reached significance. If the correlations of the random effects reached -/+1 the condition was moved from the random effect (as indicated in the results section).

Comprehension

Visual inspection of comprehension accuracy (see Figure S2.1; Table S2.1) indicates that PwNH were at ceiling. Given that running models where the majority of observations are close to the maximum value can lead to biased model estimates and inaccurate predictions (e.g., Donnelly & Verkuilen, 2017), we did not analyse this group further. Focusing on PwHL, the model included fixed effects of type (CP/CU/NP) which was treatment coded (with CP as the baseline) and load (load/no load) which was sum contrast coded (.5/-.5). The maximal model did not converge so the random effects were reduced to main effects.^[[1]](#footnote-2)^ The omnibus test revealed a main effect of type (*X*^2^(2)=14.71, *p*<0.001) with accuracy decreasing for CU compared to CP items (est.=-2.55, z=-2.73, p<0.01). Neither the main effect of load (*X*^2^(1)=0.05, *p*=0.83) nor the interaction between type and load reached significance (*X*^2^(2)=0.54, *p*=0.73). See Table S2.1 for additional information and OSF for data and analyses.


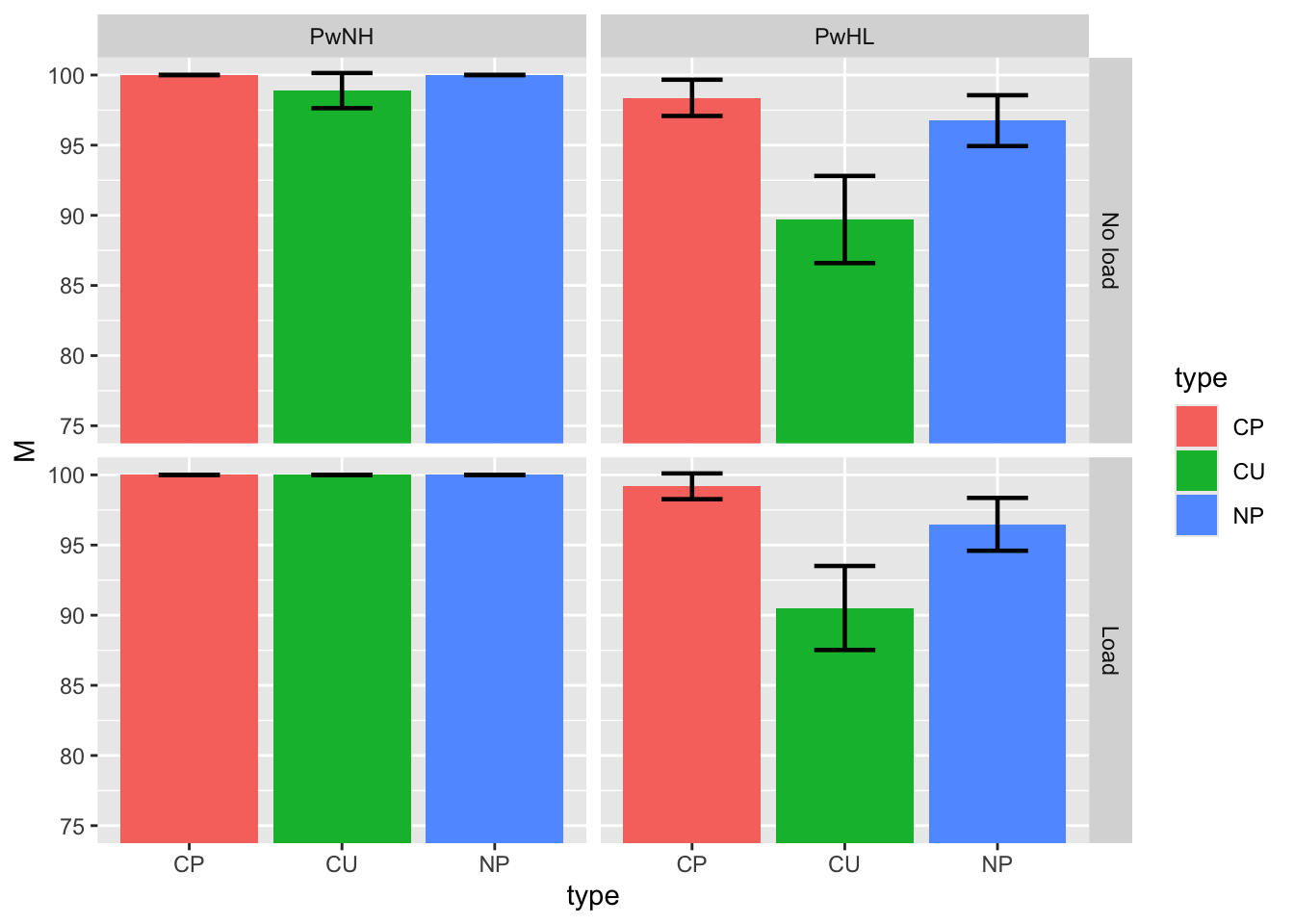


Figure S2.1. Mean comprehension accuracy by group, load, and sentence type. Error bars indication 95% confidence intervals.

Table S2.1 Comprehension accuracy model output and means

| Predictor | | Estimate | | *SE* |  | |  | | *z* | | *p* |
| --- | --- | --- | --- | --- | --- | --- | --- | --- | --- | --- | --- |
| **Intercept** | | **6.54** | | **0.81** |  | |  | | **8.09** | | **< .001***** |
| **Sentence type (CU)** | | **-2.55** | | **0.94** |  | |  | | **-2.73** | | **.006**** |
| Sentence type (NP) | | -1.16 | | 0.94 |  | |  | | -1.24 | | .215 |
| Load (Load) | | 0.72 | | 1.01 |  | |  | | 0.71 | | .480 |
| Sentence type (CU) × Load (Load) | | -0.83 | | 1.06 |  | |  | | -0.78 | | .434 |
| Sentence type (NP) × Load (Load) | | -0.84 | | 1.11 |  | |  | | -0.76 | | .449 |
| Mean (SD) comprehension accuracy (%) | | | | | | | |  | |  |  |
|  | PwNH | | | | | PwHL | | | |  |  |
| Type | No load | | Load | | | No load | | Load | |  |  |
| CP | 100 (0) | | 100 (0) | | | 98.37 (12.66) | | 99.19 (8.99) | |  |  |
| CU | 98.89 (10.5) | | 100 (0) | | | 89.7 (30.43) | | 90.51 (29.34) | |  |  |
| NP | 100 (0) | | 100 (0) | | | 96.75 (17.76) | | 96.48 (18.46) | |  |  |

Corsi

The maximal model did not converge, so the random effect of item was reduced to main effects. However, this led to -1/1 correlations between the levels of sentence type in the random effect of item, therefore sentence type was removed^[[2]](#footnote-3)^. The omnibus test revealed a main effect of group (*X*^2^(1)=5.72, *p*<0.05) with PwNH having higher accuracy than PwHL (est.=-0.97, z=-2.95, p<0.01). There was also a main effect of sentence type (*X*^2^(2)=6.32, *p*<0.05) with lower accuracy for CU compared to CP items (est.=-0.36, z=-2.74, p<0.01), and no difference between CP and NP items (est.=-0.11, z=-7.64, p=0.44). The interaction between group and sentence type did not reach significance (*X*^2^(2)=5.35, *p*=0.07). See Table S2.2 for additional information and OSF for data and analyses.

Table S2.2. Corsi accuracy model output and means

| Predictor | | Estimate | *SE* | *z* | *p* |
| --- | --- | --- | --- | --- | --- |
| Intercept | | -0.18 | 0.17 | -1.04 | .297 |
| **Group** | | **-0.97** | **0.33** | **2.95** | **.003**** |
| **Sentence type (CU)** | | **-0.36** | **0.13** | **2.74** | **.006**** |
| Sentence type (NP) | | -0.11 | 0.14 | 0.76 | .445 |
| Group × Sentence type (CU) | | 0.47 | 0.26 | 1.79 | .073 |
| Group × Sentence type (NP) | | -0.13 | 0.28 | 0.45 | .651 |
| Mean (SD) Corsi accuracy (%) | | |  |  |  |
| Type | PwNH | PwHL |  |  |  |
| CP | 55.93 (49.74) | 37.4 (48.45) |  |  |  |
| CU | 44.44 (49.78) | 35.23 (47.83) |  |  |  |
| NP | 54.44 (49.89) | 35.77 (48) |  |  |  |

*Discussion*

We first looked at comprehension accuracy (i.e., clicking correctly on the target spoken in the sentence), for which the PwNH were at ceiling, while the PwHL had higher accuracy for CP items than CU items (though note average accuracy remained at 89%). Poorer accuracy for unexpected stimuli may indicate that PwHL relied on context information to a greater extent, and overrode what was spoken with what was predicted, though more research is necessary before making broader claims. When it comes to accuracy on the Corsi task (only measurable in the load condition), recall was impacted by both hearing loss and item type, though they did not interact. PwHL showed lower overall accuracy than PwNH, supporting research that hearing loss leads can lead to greater reliance on the cognitive system to extract meaning from the speech stream, interfering with resources available with other tasks (e.g., Peelle, 2018; Pichora-Fuller et al., 2016). Additionally, both groups showed higher accuracy for CP items relative to CU items (but not for CP relative to NP items), suggesting that integrating an unexpected (but plausible) word into a constraining context also independently interferes with the ability to maintain visuo-spatial information in working memory.

See OSF for data and analyses.

References

Barr, D. J., Levy, R., Scheepers, C., & Tily, H. J. (2013). Random effects structure for confirmatory hypothesis testing: Keep it maximal. *Journal of Memory and Language*, *68*(3), 255–278. https://doi.org/10.1016/j.jml.2012.11.001

Donnelly, S., & Verkuilen, J. (2017). Empirical logit analysis is not logistic regression. *Journal of Memory and Language*, *94*, 28–42. https://doi.org/10.1016/j.jml.2016.10.005

Peelle, J. E. (2018). Listening Effort: How the Cognitive Consequences of Acoustic Challenge Are Reflected in Brain and Behavior. *Ear and Hearing*, *39*(2). https://journals.lww.com/ear-hearing/fulltext/2018/03000/listening_effort__how_the_cognitive_consequences.4.aspx

Pichora-Fuller, M. K., Kramer, S. E., Eckert, M. A., Edwards, B., Hornsby, B. W. Y., Humes, L. E., Lemke, U., Lunner, T., Matthen, M., Mackersie, C. L., Naylor, G., Phillips, N. A., Richter, M., Rudner, M., Sommers, M. S., Tremblay, K. L., & Wingfield, A. (2016). Hearing Impairment and Cognitive Energy: The Framework for Understanding Effortful Listening (FUEL). *Ear and Hearing*, *37*. https://journals.lww.com/ear-hearing/fulltext/2016/07001/hearing_impairment_and_cognitive_energy__the.2.aspx

1. Final model: (accuracy ~ participant_group*sentence_type+ ( sentence_type+load|participant) +( sentence_type+load |item), family=binomial) [↑](#footnote-ref-2)
2. Final model: (accuracy ~ participant_group*sentence_type+ ( sentence_type|participant) +( participant_group|item), family=binomial) [↑](#footnote-ref-3)
