# Supplementary material for: Involvement of executive resources in prediction: Effects of load and hearing loss in older adults
Source: Psychon Bull Rev. 2026 Apr 14;33(4):135. doi: 10.3758/s13423-026-02880-0 (PMC13079493; doi:10.3758/s13423-026-02880-0)
Supplement: Supplementary file 3 — Supplementary file3 (DOCX 248 KB) [file 13423_2026_2880_MOESM3_ESM.docx]

**DPA on CU items - analysis and results**

The third DPA investigated the timing of the integration of the unexpected target (given the context) in the 18 CU items across PwNH and PwHL in the no load and load conditions. For this we compared looks to the CU image (*tree*) vs. distractor image (*jar*) in the CU items^[[1]](#footnote-1)^. Across all comparisons the CI contained 0, this does not support reliable differences between load and/or group. See Table S3.1 for divergence times and Table S3.2 for divergence time comparisons. See Figure S3.1.


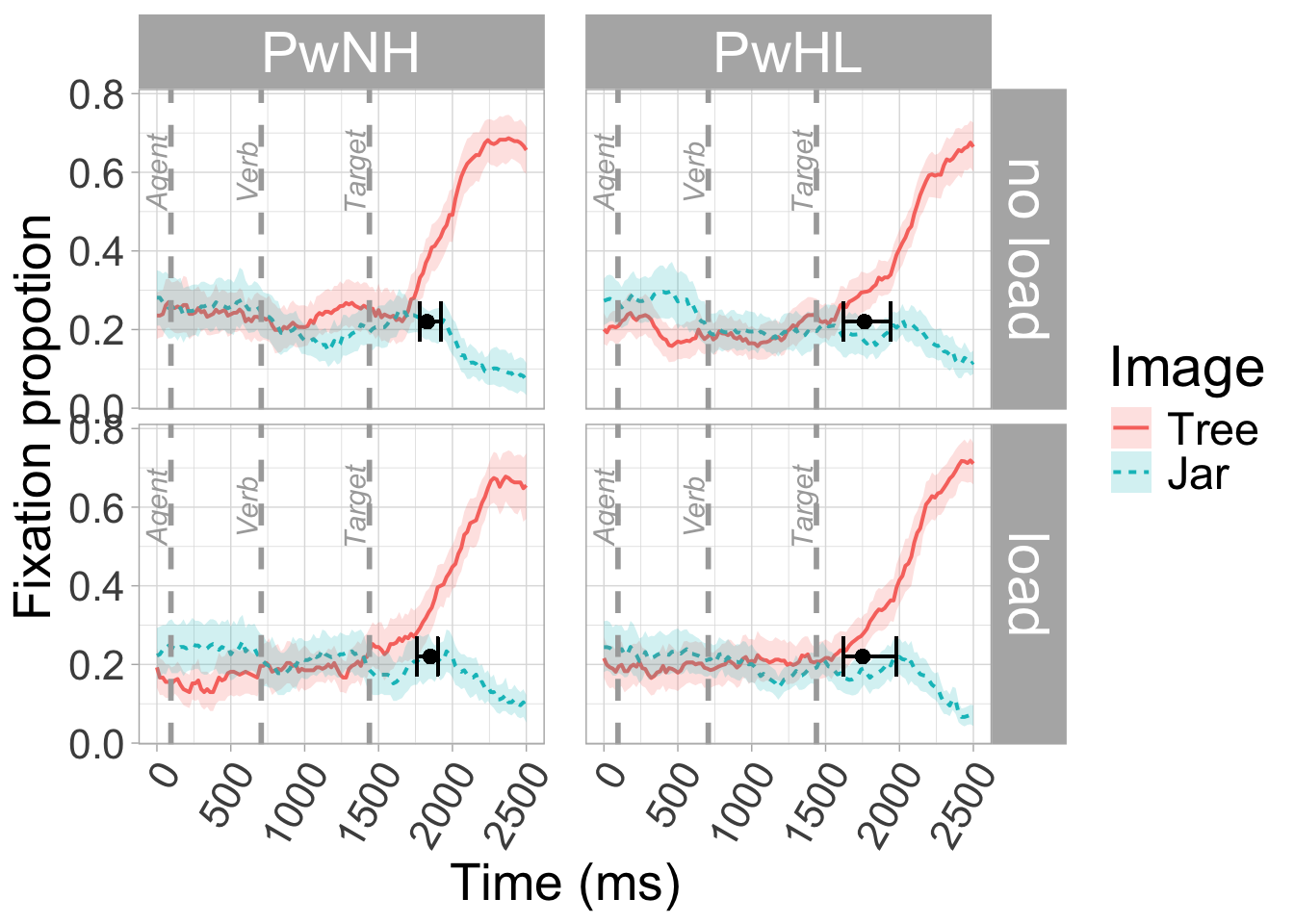


Figure S3.1. Divergence point and 95% confidence intervals superimposed on the fixation proportion of looks to the CU target and a distractor in CU items across load and group.

Table S3.1. Divergence times [95% confidence intervals] between images across load and group

| Comparison (sentence type) | Image comparison | PwNH-no load | PwNH- load | PwHL-no load | PwHL- load |
| --- | --- | --- | --- | --- | --- |
| Unexpected integration (CU) | Tree vs Jar | 1829.48 [1780,1920] | 1850.54 [1760,1900] | 1763.07 [1620,1940] | 1751.07 [1620,1980] |

Table S3.2. Difference in divergence times [95% confidence intervals] across load and group. If the CI contains zero the comparison is not considered reliably different, if the CI does not contain zero the comparisons is considered reliably different,

| Comparison (sentence type) | Image comparison | PwNH- load vs PwNH -no load | PwHL- load vs PwHL -no load | PwHL-no load vs PwNH-no load | PwHL- load vs PwNH- load |
| --- | --- | --- | --- | --- | --- |
| Unexpected integration (CU) | Tree vs Jar | 20.97 [-80,100] | -12.00 [-200,240] | -66.41 [-200,100] | -99.38 [-269,140] |

See OSF for data and analyses.

1. The DPA only allows for one divergence point, this analysis was therefore performed because after hearing *The tailor trims* looks to the *suit* should diverge from *tree*, however, after hearing *tree*, a second divergence would occur when *suit* is inhibited and *tree* is facilitated. Given the context, both *tree* and *jar* should be inhibited, however after the onset of *tree* there should be an increase in looks to *tree* but not jar. Therefore, we are able to see the timing in which previously inhibited items are integrated. [↑](#footnote-ref-1)
